# Rapid Evaluation Method to Vertical Bearing Capacity of Pile Group Foundation Based on Machine Learning

**DOI:** 10.3390/s25041214

**Published:** 2025-02-17

**Authors:** Yanmei Cao, Jiangchuan Ni, Jianguo Chen, Yefan Geng

**Affiliations:** 1 School of Civil Engineering, Beijing Jiaotong University, Beijing 100044, China; 23125978@bjtu.edu.cn (J.N.);; 2 Supervision Center of Engineering Quality, National Railway Administration of the People’s Republic of China, Beijing 100038, China

**Keywords:** pile foundation of the bridge, vertical bearing capacity, rapid evaluation, machine learning, ratio of dynamic stiffness to static stiffness

## Abstract

With the continuous increase in bridge lifespans, the rapid check and evaluation of the vertical bearing capacity for the pile foundations of existing bridges have been in greater demand. The usual practice is to carry out compression bearing tests under static loads in order to obtain the accurate ratio of the dynamic to static stiffness. However, it is difficult and costly to conduct in situ experiments for each pile foundation. Herein, a rapid evaluation method to measure the vertical bearing capacity of bridge pile foundations is proposed. Firstly, a 3D-bearing cap–pile group–soil interaction model was established to simulate a bearing test of a pile foundation that was subject to static loads and dynamic loads, and then the numerical results were validated by in situ dynamic and static loading tests on an abandoned bridge pier with the same pile group foundation; the dataset for machine learning was constructed using the numerical results, and finally, the bearing capacity of the pile foundation could be predicted rapidly. The results show the following outcomes: the established numerical model can effectively simulate dynamic and static loading tests of pile foundations; the intelligent prediction model based on machine learning can predict the ratio of static stiffness to dynamic stiffness and can thus rapidly evaluate the vertical residual bearing capacity and the designed ultimate loading capacity, allowing for the nondestructive testing and evaluation of the pile foundations of existing bridges.

## 1. Introduction

Pile foundations are extensively used in various types of infrastructure owing to their exceptional benefits [1]. The characteristics of high-speed railways, such as the high speed, high user comfort, high safety, and high-density continuous operation, necessitate strict requirements for the cumulative settlement and vertical bearing capacity of bridge pile foundations [2]. Therefore, establishing an effective and rapid evaluation method to determine the bearing capacity of pile group foundations is very significant.

In recent decades, many evaluation methods, including on-site testing [3], theoretical analysis [4], and numerical calculations [5,6], have been proposed. The input parameters derived from in situ soil tests (such as the Standard Penetration Test (SPT) and the Cone Penetration Test (CPT)) are often preferred over the input parameters derived from laboratory soil tests, which may introduce errors due to soil sample disturbance [7]. The mechanical impedance method is becoming one of the most commonly used methods for pile foundation detection [8], which entails the evaluation of the bearing capacity of pile foundations through establishing the dynamic-to-static contrast coefficient between the dynamic and static stiffness of the pile foundation. In principle, static stiffness can be measured by compression bearing tests under static loads. However, it is difficult to carry out compressive static load tests when a bridge is in service, and the pile group foundation is concealed below ground level. Moreover, conducting a large number of in situ experiments is time-consuming and costly. Even if the static stiffness can be measured, due to the large differences in geological conditions in different regions (e.g., frozen soil [9], collapsible loess [10], saline soil [11], soft soil [12], etc.), there are often large differences in the value of the dynamic-to-static stiffness contrast coefficient. Even the evaluation of the bearing capacity of pile foundations of the same kind under the same soil conditions often produces large errors due to the uncertainty of the test.

With the continuous development of artificial intelligence and computer technology, there is a new trend in predicting the characteristic parameters of pile foundations through the use of computer algorithm programs and the training of machine learning models on comprehensive datasets [13]. With a large amount of existing test data containing multiple pile properties (e.g., length, diameter, material properties, etc.) and loading conditions (e.g., the type of load, the load duration, the properties of the surrounding soil, etc.), it is possible to more efficiently predict and understand the complex non-linear relationships between the pile’s load-bearing behavior and the input parameters [14]. This approach offers several advantages over traditional in situ load testing, including enhanced cost-effectiveness, greater simplicity, and improved reliability [15,16,17]. Momeni [18] used a pile driving analyzer to measure the bearing capacity of piles and developed a prediction model to measure the vertical bearing capacity of pile foundations based on genetic algorithm optimization technology. The test results showed that the accuracy coefficient and the mean square error were 0.990 and 0.002, respectively, which proves the reliability of machine learning in this field. Román Quevedo-Reina [19] reproduced the equivalent linear stiffness of pile foundations in heterogeneous soil based on a surrogate model of an artificial neural network (ANN). Cheng [20] proposed a practical machine learning method using random forest (RF) optimized by Bayesian optimization (BO) and particle swarm optimization (PSO) to improve the prediction accuracy and better adapt to different geological environments to predict the driving performance parameters of piles (i.e., maximum compressive stress, maximum tensile stress, and blow per foot). Wang [21] used two meta-heuristic algorithms, YYPO (Yin-Yang-pair Optimization) and SA (Simulated Annealing), to fully explore the performance of MLS-SVR and to predict the drivability of piles.

Considering the uncertainty of multiple parameters, Wen [22] introduced the artificial intelligence model and proposed a fast analysis and calculation method for the reliability of bridge foundation piles based on settlement control. This method was used to study the reliability of foundation piles based on settlement control in a bridge under construction. Liao [23] studied the pile type, pile length, pile reinforcement, and other aspects. Under the premise of meeting the safety and quality requirements of pile foundation design, the optimization calculation method of a pile foundation was put forward, and the fine calculation of a pile foundation of a railway bridge was realized.

Compared to traditional methods, machine learning techniques excel in processing large datasets and identifying complex patterns. Nejad [16] developed a learning model based on an artificial neural network to predict the bearing characteristics of piles based on 500 sets of cone penetration test results and compared it with the results of a compressive static load test to obtain a more accurate *Q-S* curve for predicting the bearing characteristics of piles. Tan Nguyen [24] pioneered the application of advanced metaheuristic algorithms, most notably 3mSOS. Xu [25] presented a novel approach combining binocular vision with YOLOv8, a state-of-the-art object detection method, to enhance the efficiency, safety, and reliability of underwater pile foundation concrete defect detection. Medina [26] employed the regression algorithm to establish an analysis model of pile-layered soil interaction to evaluate the influence of layered homogeneous soil and subsequently proposed an empirical formula for the bearing capacity of pile foundations. Zhang [27] developed a Bayesian network algorithm to establish a deviation factor training prediction model based on field static load test data and finite element modeling simulation data of pile foundations in Shanghai. They analyzed the factors that caused large deviations between the simulation model and the test data, providing a reference for pile foundation design.

To understand the influence of the parameters on the prediction accuracy, Deng [28] utilized kernel ridge regression (KRR) and multi-layer perceptron (MLP) algorithms to establish vertical and horizontal load-bearing capacity prediction models of single piles in a salt marsh environment, respectively. Hu [29] proposed a data-driven white-box Bayesian network model and expert system to identify the key factors affecting the performance or resistance of bridge piles, capture the causal (direct and indirect) relationship between different variables affecting the resistance of bridge piles, and predict the resistance of bridge piles to support decision making in bridge design and construction. Cao [30] developed and validated a novel data-driven multivariate neural network inference model (IMNNIM) to predict the axial bearing capacity of piles. Chen [31] predicted the bearing capacity of piles developing several intelligent models, i.e., neuro-genetic, neuro-imperialism, genetic programming (GP), and an artificial neural network (ANN). Harandizadeh [32] developed a novel artificial intelligence prediction model (ANFIS-GMDH-PSO) for pile bearing capacity, demonstrating superior performance compared to the ANN and FPNN-GMDH models.

Due to the varying accuracies of different prediction methods employing different machine learning algorithms, Kardani [33] employed six machine learning algorithms—decision tree, k-nearest neighbors, multi-layer perceptron, random forest, support vector regression, and extreme gradient boosting to model the load-carrying capacity of piles in cohesionless soils and optimized the hyperparameters of these algorithms using particle swarm optimization. Nguyen [34] presented a hybrid model combining the extreme gradient boosting machine (XGBoost) and the whale optimization algorithm (WOA) to predict the bearing capacity of concrete piles. Amjad [35] developed a novel model for predicting bearing capacity using the extreme gradient boosting (XGBoost) algorithm.

In order to solve the difficulty of carrying out compressive static load tests on existing bridges, this paper proposes a rapid prediction method for the dynamic-to-static stiffness contrast coefficient to evaluate the vertical bearing capacity of pile group foundation of a bridge. The developed method combines an in situ experiment, a three-dimensional numerical analysis of the platform–pile–soil interaction, and machine learning, offering a novel approach for pile foundation survey evaluation and design.

## 2. Methodology

The vertical bearing capacity of a pile group foundation is closely related to the dynamic stiffness or the dynamic-to-static contrast coefficient, as determined by the low-strain pile–soil dynamic impedance method [36]. This research aims to determine the relationship using a machine learning technique.

Given the impracticality of obtaining a machine learning model’s training dataset solely through experimentation, a numerical model of a bearing platform–pile group–soil interaction was established. The effectiveness of the numerical model was validated by an in situ experiment. Subsequently, the numerical model simulated a large number of dynamic loading tests and compression static tests, and the influence of the pile parameters on the vertical dynamic and static stiffness of the structure was studied by the numerical model, considering different types of piles and environmental effects.

Next, using the machine learning algorithm, learning and training were conducted on the samples of the dynamic stiffness values, static stiffness values, and dynamic-to-static contrast coefficients.

Finally, the prediction functions were obtained, which can be further applied to rapidly evaluate the vertical residual bearing capacity, the design ultimate bearing capacity, and the design initial dynamic stiffness of the structure.

**Figure 1:**
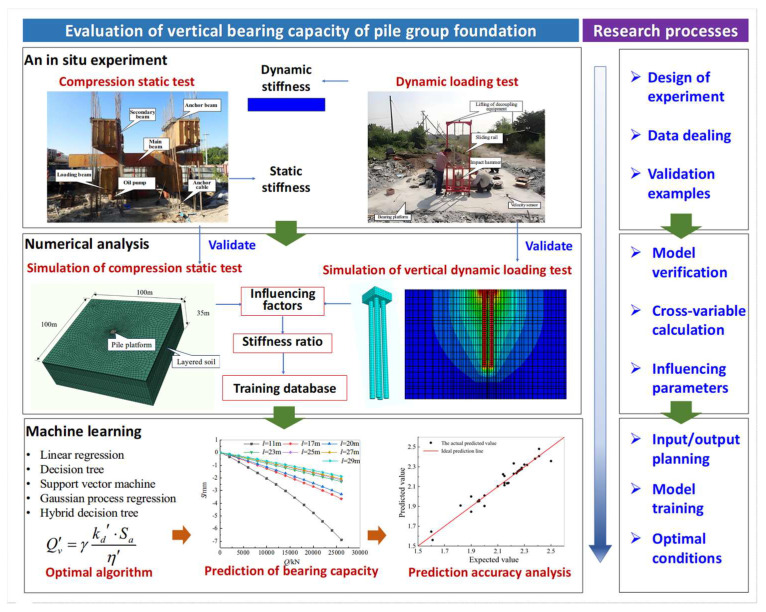
Framework of the proposed method.

The framework of the rapid evaluation method is illustrated in Figure 1.

## 3. Numerical Simulation and Experimental Verification of Nondestructive Pile Foundation Testing

### 3.1. Three-Dimensional Numerical Model of Pile Cap–Pile Group–Soil Interaction

**Figure 2:**
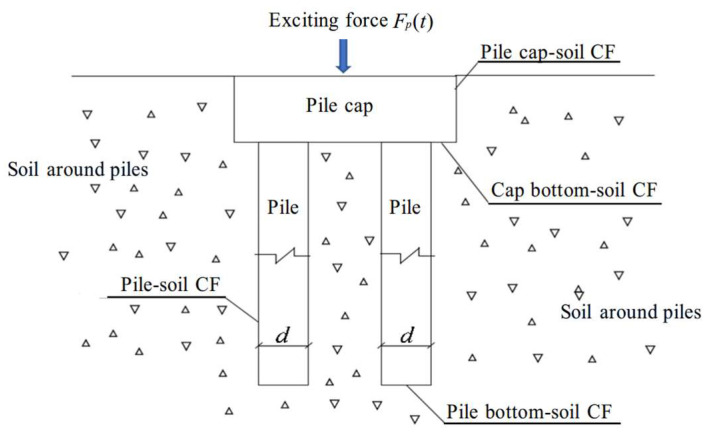
Sketch of contact relationship of pile–soil contact model.

The mechanical impedance method requires the application of an instantaneous excitation force to the pile cap surface to measure the resulting surface vibration response. The interaction between the pile cap, pile foundation, and layered subsoil introduces contact non-linearities into the system’s dynamic response. These interactions, which occurred across various contact interfaces (CFs), are illustrated in Figure 2.

**Figure 3:**
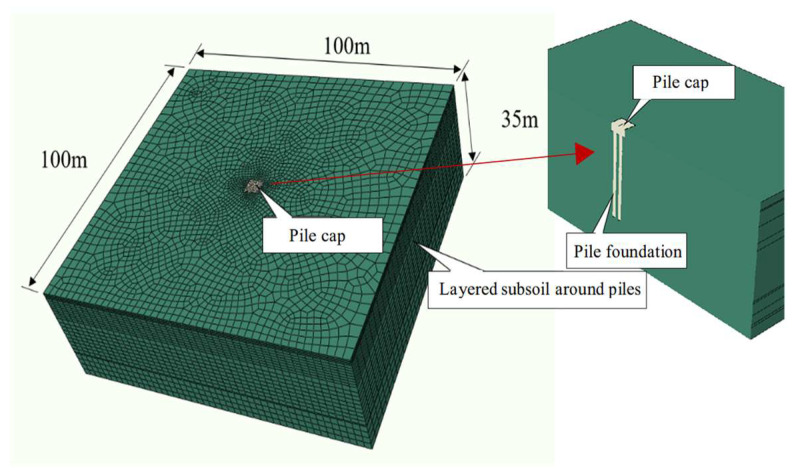
Three-dimensional numerical model of bearing platform–pile group–soil.

**Figure 4:**
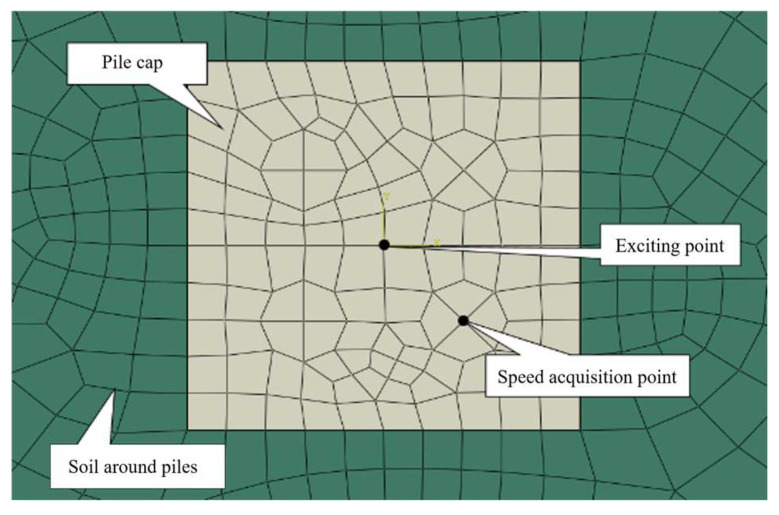
Setting of exciting point and sampling point.

Given the complex mechanical behavior of soil, influenced by factors such as the density, loading conditions, and void content, an ABAQUS finite element model was developed to simulate the dynamic interaction between the cap, pile group, and soil (Figure 3). Figure 4 illustrates the arrangement of the excitation and measurement points in the numerical model.

**Figure 5:**
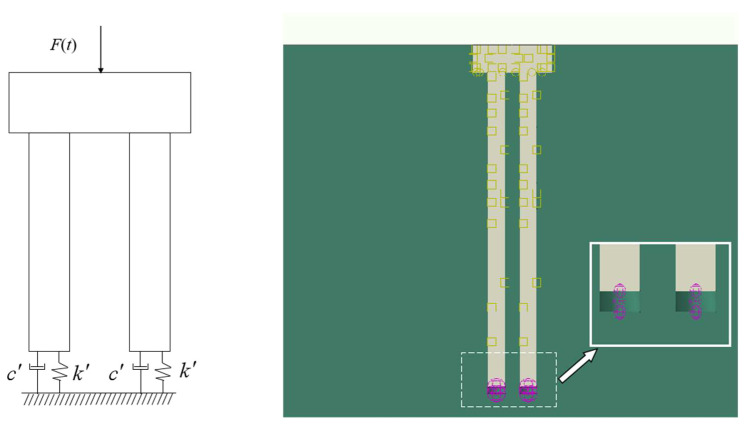
Sketch of elastic contact between pile bottom and subsoil.

For accurate calculations, an eight-node linear hexahedral element was used for the foundation soil. A graded mesh, refined near the pile cap and progressively coarser away from it, was used, as shown in Figure 3. The finite element mesh featured 30 divisions for the 100 m region and 15 divisions for the 35 m region. The mesh employed a linear order and a reduced integration scheme with hourglass control, and it was finely meshed around the cap. Rigid contact was modeled between the cap and pile, while frictional contact was used at the interfaces between the cap/soil and pile/soil, to capture the interaction of different components. Vertical interaction was assumed between the pile base and the surrounding soil under the excitation load. Due to the significant difference in stiffness between the pile and soil and the small relative displacements, a viscoelastic interaction was modeled. Spring-dashpot elements were incorporated between the pile base and the surrounding soil to simulate this viscoelastic behavior (Figure 5). Spring and damping coefficients at the pile base–soil interface were determined using elastic half-space theory [37].

(1) $$k^{\prime }=4Gr/(1-\nu ),$$

(2) $$c^{\prime }=3.4r^{2}\sqrt{\rho^{\prime }G}/(1-\nu ),$$

where *G*, *ν,* and $\rho^{\prime }$ are the shear modulus, Poisson ’s ratio, and density of the soil at the pile base, respectively, and *r* is the pile radius.

The dynamic equation of cap–pile group–soil system can be written as

(3) $$M\ddot{u}+C\dot{u}+Ku=F_{P},$$

in which u, $\dot{u}$, and $\ddot{u}$ are the displacement, velocity, and acceleration vectors, respectively; $F_{P}$ is the external excitation vector; M is the mass matrix; K is the stiffness matrix; C=αM+βK is a Rayleigh damping matrix, with *α* and *β* being proportional constants related to the structure’s natural frequencies and damping ratios.

If the dynamic equilibrium condition is satisfied at time *t*,

(4) $$M\ddot{u}=F_{P}-I,$$

where $I=C\dot{u}+Ku$ denotes the nodal internal force of the system.

At time *t*, the initial acceleration of the incremental step is

(5) $${\left.\ddot{u}\right|}_{t}=M^{-1}{\left.\left(F_{P}-I\right)\right|}_{t}.$$

Assuming constant acceleration over a time increment, the velocity increment is calculated using the central difference method and added to the velocity at the midpoint of the previous time increment. Thus, the velocity of $t+\frac{1}{2}\Delta t$ at the midpoint of the current time increment is

(6) $${\left.\dot{u}\right|}_{t+\frac{1}{2}\Delta t}={\left.\dot{u}\right|}_{t-\frac{1}{2}\Delta t}+\frac{{\left.\Delta t\right|}_{t+\frac{1}{2}\Delta t}+{\left.\Delta t\right|}_{t}}{2}{\left.\ddot{u}\right|}_{t},$$

where Δt is the length of the incremental step.

The displacement at the end of the incremental step (i.e., at time t+Δt) can be obtained by integrating the velocity:

(7) $${\left.u\right|}_{t+\Delta t}={\left.u\right|}_{t}+\Delta t{\left.u\right|}_{t+\frac{1}{2}\Delta t}.$$

Using the displacement from Equation (7), the strain is calculated, and subsequently, the stress is determined using the stress–strain constitutive relationship. This allows for the calculation of nodal internal forces $I_{t+\Delta t}$. The process then proceeds to the next incremental step, repeating the integration calculation until the analysis is complete.

### 3.2. Calculation of Vertical Bearing Capacity of Pile Foundation

When performing a static load test on a pile foundation, a fitting curve is generated relating the cumulative load (*Q*) at the pile head to the resulting cumulative settlement. The slope of the linear portion, representing the elastic stage of this curve, corresponds to the overall static stiffness ($k_{s}$) of the pile–soil system. Therefore, the vertical bearing capacity of a pile foundation $Q_{v}$ is estimated by

(8) $$Q_{v}=k_{s}S_{a},$$

where $S_{a}$ is the allowable settlement of the pile foundation (unit: mm).

However, because it is difficult to subject many existing bridges in service to a compressive static load test on the pile foundation, $k_{s}$ is not easily obtained directly. In engineering practice, the dynamic load test of the cap–pile group is often performed using the mechanical impedance method to determine the dynamic stiffness ($k_{d}$) of the pile foundation. A comparison coefficient between the dynamic and static stiffness is estimated, and then the vertical bearing capacity of the pile foundation is derived as

(9) $$Q_{v}=\frac{k_{d}S_{a}}{\eta }.$$

In the dynamic load test of a pile foundation, the velocity admittance function of the pile–soil system is defined as

(10) $$Y(f)=\frac{\hat{\dot{u}}(f)}{{\hat{F}}_{P}(f)},$$

where *f* is the excitation frequency, and ${\hat{F}}_{P}(f)$ and $\hat{\dot{u}}(f)$ are the excitation force and velocity response in the frequency domain, respectively, which can be obtained by the Fourier transform of $F_{P}(t)$ and $\dot{u}(t)$.

According to the Technical Code for Detection of Building Piles [38], when the excitation frequency is low, the dynamic stiffness ($k_{d}$) of the pile–soil system under different excitation frequencies can be expressed as

(11) $$k_{d}(f)=\frac{2\pi f}{\left|Y(f)\right|}.$$

### 3.3. Experimental Verification of Numerical Analysis Model

**Figure 6:**
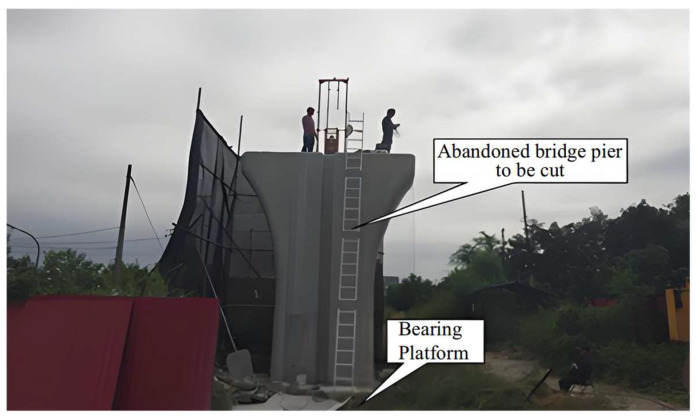
The experiment site and abandoned bridge pier to be cut.

To validate the presented numerical model, experimental data from existing studies [39,40] were utilized. These tests were conducted on an abandoned pier of the Beijing subway line in the western suburbs (Figure 6), which was cut during the compression bearing test and dynamic test. The pier foundation comprised four friction piles constructed of C30 concrete, with a nominal length of 23 m and a diameter of 1200 mm.

**Figure 7:**
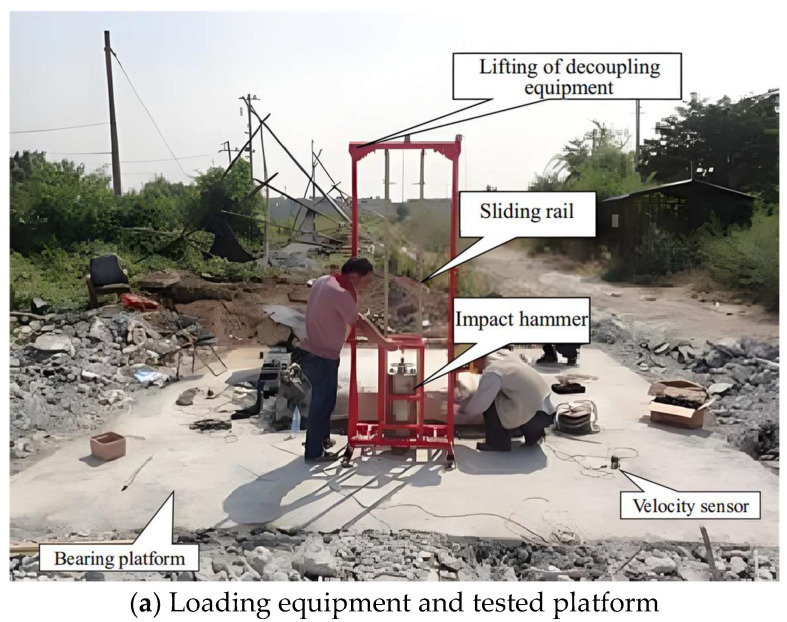

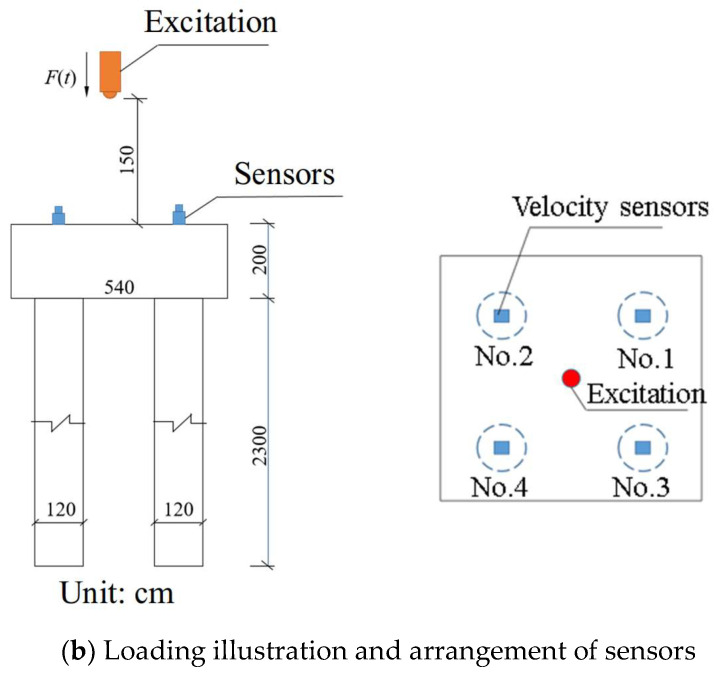
Test arrangement: (**a**) Photos of loading equipment and tested platform; (**b**) Illustration of loading and sensor arrangement.

An impact hammer was developed to excite the vibration of the pile foundation (Figure 7a), and four velocity sensors were laid out on the corner of the bearing platform (Figure 7b).

**Figure 8:**
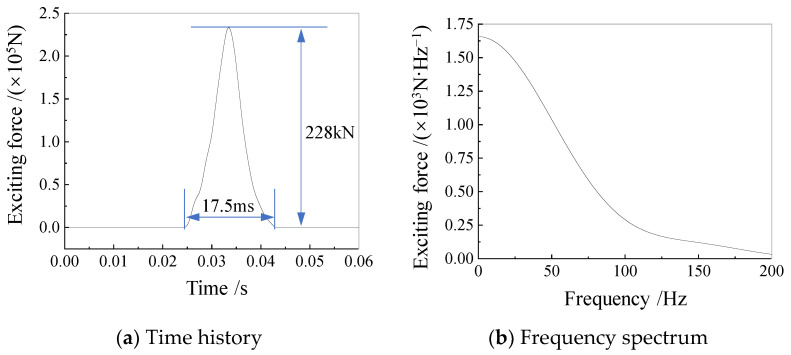
Loading excitation of on-site test for dynamic stiffness: (**a**) Time history of the applied excitation; (**b**) Frequency spectrum of the applied excitation [19].

During the compression static test, a 13-stage loading protocol was adopted, reaching a maximum load of 228,000 kN. During the dynamic stiffness test, the loading equipment provided a pulse excitation force, for 17.5 ms, which was applied to the top center of the pile cap (Figure 8).

A three-dimensional finite element numerical model of the pile cap–pile group–soil interaction was developed. The soil parameters are presented in Table 1. Modal analysis of the system was first performed. The fundamental circular frequency was calculated as 15.62 rad/s. The corresponding circular frequency corresponding to the maximum vertical vibration mode participation factor was 20.64 rad/s. The damping ratio of the pile cap–pile group system is typically 0.05, and the soil damping ratio typically ranges from 0.02 to 0.03. Therefore, an overall damping ratio of 0.03 was adopted for the pile cap–soil system. Then, the Rayleigh damping coefficients in Equation (3) were obtained as α=0.533 and β=0.0016.

(1) Verification of the static stiffness prediction for bridge pile foundations

**Figure 9:**
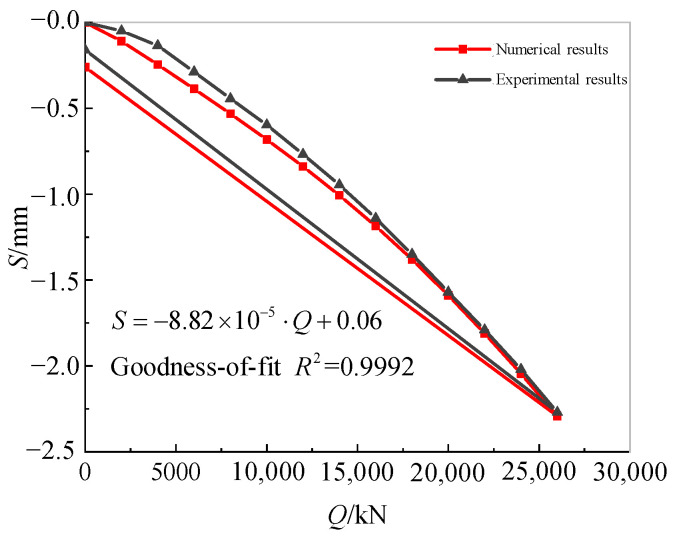
Comparison of numerical and experimental *Q*-*S* curves.

Figure 9 compares the *Q*-*S* curves for the cumulative load–settlement obtained from the compressive static load test and the numerical simulation. It can be seen that the numerical simulation results exhibited a similar trend and comparable magnitude to the experimental *Q*-*S* curves. The overall agreement was satisfactory. The numerical simulation underestimated the cumulative settlement at the first five load levels. This discrepancy is likely attributable to the idealized nature of the Mohr–Coulomb plastic model employed in ABAQUS. Prior to plastic yielding, the deformations and settlements under load were generally consistent.

The numerical model’s *Q-S* curve was fitted linearly, yielding a vertical static stiffness value of $1.13\times 10^{10}\;N/m$ for the cap–pile group. This value closely approximated the experimental value of $1.11\times 10^{10}\;N/m$.

(2) Verification of bridge pile foundation dynamic stiffness

**Figure 10:**
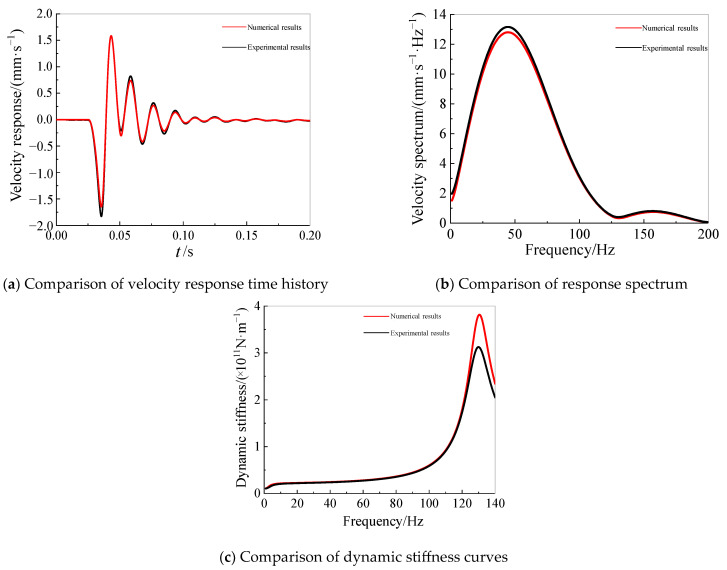
Comparison of numerical and experimental results.

Figure 10 shows the comparison between the numerical simulation results and the dynamic load test results of the pile foundation. It can be seen that the velocity time histories and frequency spectra from the pile cap sensors generally matched. In the frequency range below 100 Hz, the dynamic stiffness values from the numerical simulation aligned well with the experimental values.

Therefore, the numerical simulation method presented in this paper is accurate and effective for determining the static and dynamic stiffness of pile foundations and can be used to simulate and analyze nondestructive pile foundation testing.

## 4. Machine Learning Prediction of Pile Foundation Dynamic and Static Stiffness

### 4.1. Cross-Application of Numerical and Machine Learning Models

**Figure 11:**
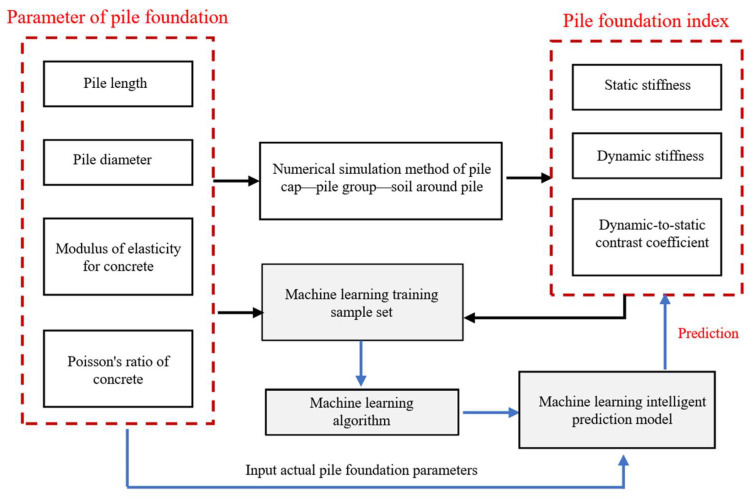
Cross-application of numerical and machine learning models.

Figure 11 illustrates the cross-application of the numerical analysis model and machine learning. The input data of the machine learning model are the pile length, pile diameter, elastic modulus, and Poisson’s ratio of the pile group foundation, and the output data are the static stiffness, dynamic stiffness, and the dynamic-to-static contrast coefficient.

Using the developed numerical model of the bearing platform–pile group–soil interaction, a dataset of vertical static and dynamic stiffness and dynamic-to-static contrast coefficients was generated by varying the pile foundation parameters (length, diameter, elastic modulus, Poisson’s ratio). According to the results from the on-site experiment, the pile length of the pile group foundation ranged from 17 m to 29 m, and the pile diameter ranged from 0.8 m to 1.6 m. Since the environmental factors (such as thermal effects, etc.) also have an impact on the bridge structure, the elastic modulus and the Poisson’s ratio of concrete material were also taken as the variables for the machine learning. The variation range of the elastic modulus was 28,000–34,500 MPa, and that of the Poisson ratio was 0.15–0.2. By taking into account a large number of different situations, the output results of the finite element models were obtained as the input data for the machine learning.

Then, five machine learning algorithms (linear regression, decision tree, support vector machine, Gaussian process regression, and hybrid tree) were trained and applied using MATLAB. Finally, the performance of each algorithm was evaluated using four metrics: the root mean square error (RMSE), the coefficient of determination (R^2^), the mean square error (MSE), and the mean absolute error (MAE). A higher prediction accuracy corresponds to an R^2^ value closer to 1 and smaller values of the RMSE, MSE, and MAE.

### 4.2. Machine Learning Prediction of Vertical Static Stiffness

The evaluation metrics for the prediction results from the five machine learning algorithms, after training and optimization, are presented in Table 2.

**Figure 12:**
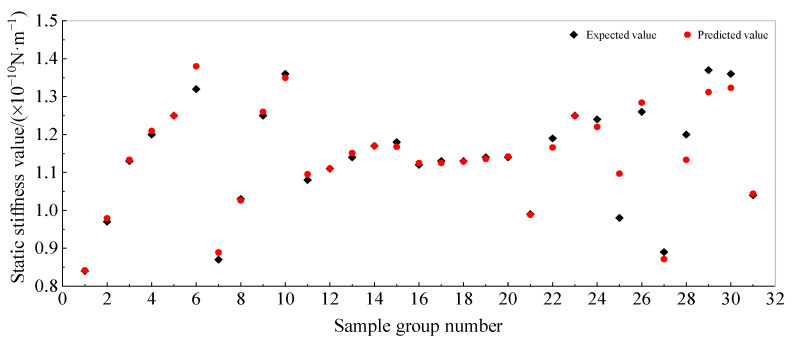
Prediction results of vertical static stiffness of pile foundation.

Table 2 shows that the Gaussian process regression (GPR) algorithm using the ARD Matern 3/2 kernel function achieved the highest prediction accuracy. Figure 12 illustrates the comparison between the predicted and actual vertical static stiffness values for this method. The plot demonstrates a close agreement between the predicted and expected values, indicating high accuracy in the predictions.

**Figure 13:**
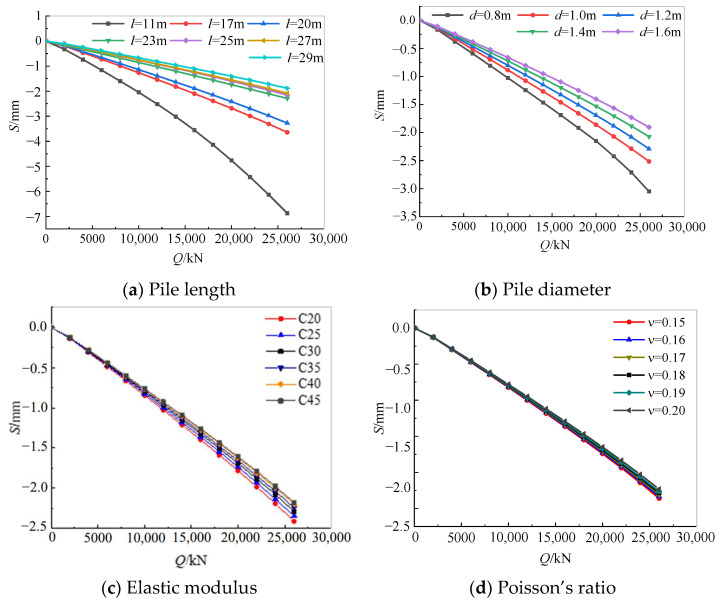
Influence factor analysis of vertical static stiffness of pile foundation.

Figure 13 shows the influence of the pile length, pile diameter, elastic modulus, and Poisson’s ratio of the pile group foundation on the *Q-S* curve and static stiffness. It can be seen that under the layered soil conditions in the tested field, the vertical static stiffness of the structure was directly proportional to the pile length, pile diameter, pile elastic modulus, and Poisson’s ratio. The bearing capacity of the structure increased significantly as the pile length increased from 11 m to 23 m, and the increase slowed down after the pile length reached 23 m. It is recommended that the designed pile length in this area should be no less than 17 m. The bearing capacity of the structure also increased significantly as the pile diameter increased from 0.8 m to 1.2 m. The vertical static stiffness value of the structure is less sensitive to changes in the elastic modulus and Poisson’s ratio of the concrete.

### 4.3. Machine Learning Prediction of Vertical Dynamic Stiffness

**Figure 14:**
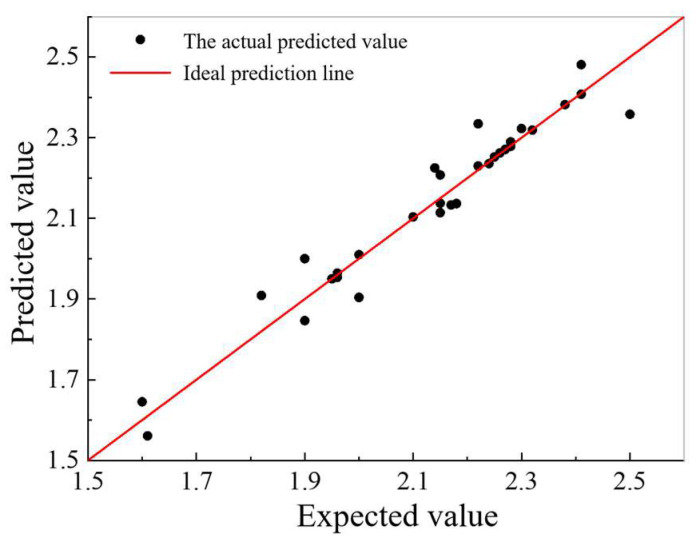
Prediction results for the vertical dynamic stiffness of pile foundations.

Table 3 presents the evaluation metrics for the prediction results of all the regression algorithms using a 20 Hz excitation frequency for the dynamic stiffness. The results indicated that the Gaussian process regression (GPR) algorithm employing the Matern 5/2 kernel function, yielded the highest prediction accuracy. Figure 14 displays the comparison between the predicted and actual dynamic stiffness values. The figure shows a generally close agreement between the predicted and expected values, highlighting the high accuracy of the model.

**Figure 15:**
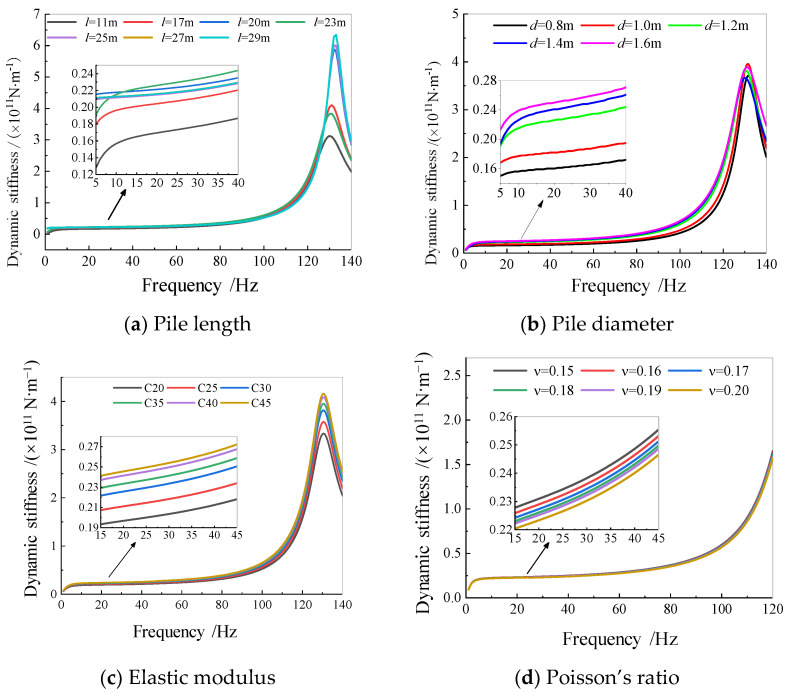
Influence factor analysis of vertical dynamic stiffness of pile foundation.

Figure 15 shows the influence of the pile length, pile diameter, elastic modulus, and Poisson’s ratio of the pile group foundation on the dynamic stiffness. It can be seen that under the layered soil conditions in the tested field, the vertical dynamic stiffness of the structure in the high-frequency range of 130–140 Hz increased sharply with the increase in the pile length, and the change was drastic. However, with the increase in the pile diameter, the vertical dynamic stiffness value increased sharply in the medium–high frequency range of 100–120 Hz, and the change in the high-frequency range was not obvious. The changes in the elastic modulus and Poisson’s ratio of the pile body had no significant impact on the medium–high and high-frequency ranges of the vertical dynamic stiffness, indicating that the high-frequency range vertical dynamic stiffness value is not suitable for evaluating the vertical bearing capacity of pile foundations.

### 4.4. Learning and Prediction of the Dynamic-to-Static Stiffness Contrast Coefficient of Pile Foundations

The K-fold cross-validation method was employed for machine learning on the dynamic-to-static contrast coefficient, using a dataset generated by various parameter combinations. The pre-training of the model used a K value of 8.

Using a 20 Hz excitation frequency as an example, Table 4 presents the accuracy comparison of five machine learning algorithms for predicting the dynamic-to-static stiffness contrast coefficients.

**Figure 16:**
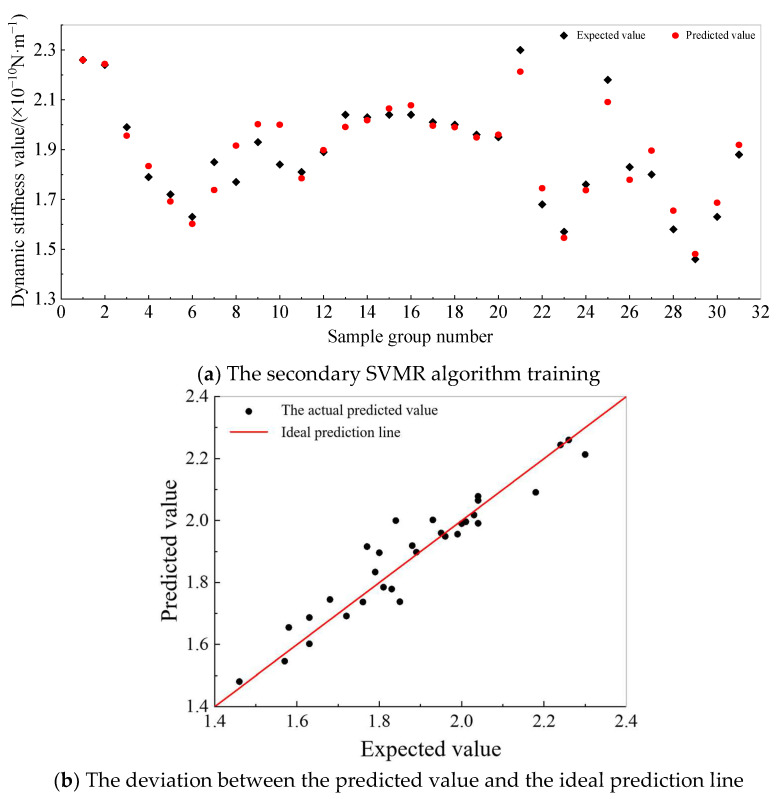
Prediction results of dynamic-to-static stiffness ratio of pile foundation.

The results from Table 4 indicate that the support vector regression (SVR) model with a quadratic kernel function exhibited the highest predictive accuracy. Figure 16 visually compares the predicted and actual values, demonstrating an improved training performance and higher accuracy for this model.

Through 31 different parameter combinations, the dynamic-to-static contrast coefficient distribution of the platform–group pile structure was obtained for the specified field. The results found that the coefficient value was inversely proportional to the pile length, and it first increased and then decreased with the increase in the pile diameter, reaching the maximum coefficient of 1.99 at a pile diameter of 1.2 m. The dynamic-to-static contrast coefficient was directly proportional to the elastic modulus of the pile concrete. There was a significant increase from C25 concrete to C35 concrete; further increasing the elastic modulus of the concrete did not result in significant changes in the coefficient. The coefficient was inversely proportional to the Poisson’s ratio, but it was not sensitive to changes in the Poisson’s ratio.

## 5. Simulation and Experimental Verification of Nondestructive Testing Methods for Pile Foundations

### 5.1. Evaluation of the Vertical Residual Bearing Capacity of a Pile Foundation

Using the previously developed machine learning model for predicting the dynamic-to-static contrast coefficient of the pile foundation and incorporating Equation (9), the vertical residual bearing capacity of the target cap–pile group structure (with soil parameters similar to those surrounding the existing piles) was evaluated:

(12) $$Q_{v}^{\prime }=\gamma_{1}\frac{k_{d}^{\prime }\cdot S_{a}}{\eta^{\prime }},$$

where $Q_{v}^{\prime }$ is the vertical residual bearing capacity of the structure to be evaluated; $k_{d}^{\prime }$ is the dynamic stiffness value measured in the field of the pile foundation to be evaluated (corresponding to different excitation frequencies); $\eta^{\prime }$ is the predicted value of the dynamic-to-static contrast coefficient from machine learning corresponding to the parameters of pile foundation to be evaluated; $\gamma_{1}$ is the safety factor.

Using a 20 Hz excitation frequency as an example, since the R^2^ index of the prediction model accuracy of the dynamic and static stiffness contrast coefficient is 0.92, and it is recommended to set 5% redundancy during the evaluation, the value of the $\gamma_{1}$ coefficient is

(13) $$\gamma_{1}=0.92÷(1+5\%)=0.88.$$

For example, considering a pile foundation with characteristics similar to those in Table 1, the foundation is a 2 × 2 friction pile with a length of 35.3 m and a diameter of 1.5 m. The concrete strength grade is C25, the Poisson’s ratio is 0.18, the design allowable bearing capacity is 22,600 kN, and the design allowable settlement is 3 mm. The dynamic stiffness of the evaluated cap–pile group structure was measured in situ, yielding a value of $k_{d}^{\prime }=2.1\times 10^{10}\;N/m$ at an excitation frequency of 20 Hz. Inputting the pile foundation’s parameters into the machine learning model for predicting the dynamic-to-static stiffness contrast, the predicted value was 1.2663. This value, according to Equation (12), yields the residual bearing capacity:

$$Q_{v}^{\prime }=0.9\times \frac{2.1\times 10^{10}\times 3\times 10^{-3}}{1.2663}=4.47\times 10^{4}\;\mathrm{kN}.$$

Therefore, the residual bearing capacity of the pile foundation is predicted to be 1.98 times the design allowable bearing capacity.

### 5.2. Evaluation of Ultimate Vertical Bearing Capacity for Designing a Pile Foundation

Using the machine learning model for static pile stiffness developed in the previous section, the ultimate vertical bearing capacity of the pile cap–pile group can be estimated for soil conditions similar to those at the test site:

(14) $$P^{\prime }=\gamma_{2}\cdot k_{s}^{\prime }\cdot S_{a},$$

where $P^{\prime }$ is the predicted value of the vertical design ultimate bearing capacity of the structure to be evaluated; $k_{s}^{\prime }$ is the machine learning static stiffness prediction value corresponding to the pile foundation parameters to be evaluated; $\gamma_{2}$ is the safety factor.

In this example, the static stiffness prediction model exhibited 95% accuracy. A 5% redundancy factor is recommended for evaluation, resulting in a coefficient value of $\gamma_{2}$:

(15) $$\gamma_{2}=0.95÷(1+5\%)=0.91.$$

Similarly, using the machine learning model for the pile foundation static stiffness, a predicted static stiffness value of 1.8 was obtained for the design. This corresponds to a predicted reference value for the vertical design ultimate bearing capacity of

$$P^{\prime }=0.91\times 1.8\times 10^{10}\times 3\times 10^{-3}=4.91\times 10^{7}\;N$$

Therefore, the ultimate bearing capacity is predicted to be 2.18 times the allowable bearing capacity.

## 6. Discussion and Conclusions 

To address the challenges of accurately determining dynamic-to-static stiffness ratios and performing static load compression tests on existing bridge pile foundations, this paper proposes a method for the rapid prediction of these ratios and the vertical bearing capacity evaluation. The following conclusions can be drawn:

(1) The numerical analysis model and machine learning algorithms can be integrated to develop a prediction model for the static, dynamic, and dynamic-to-static stiffness ratios of bridge pile foundations, in which numerical simulation provides an approach to forming a training dataset for machine learning, where field testing is impractical.
(2) The vertical residual and ultimate bearing capacities of pile groups can be quickly assessed by using only dynamic stiffness test data and machine learning techniques.
(3) The dynamic-to-static contrast coefficient is inversely proportional to the pile length, and it first increases and then decreases with the increase in the pile diameter, reaching a maximum coefficient of 1.99 at a pile diameter of 1.2 m.
(4) The dynamic-to-static contrast coefficient is directly proportional to the elastic modulus of the pile concrete. A significant increase is observed when transitioning from C25 to C35 concrete, after which, further increases in the elastic modulus have a negligible effect on the coefficient. Conversely, while the coefficient is inversely proportional to the Poisson’s ratio, this relationship is not sensitive to variations in the Poisson’s ratio.

However, the dynamic-to-static stiffness ratio of pile foundations is significantly affected by geological conditions and prevailing environmental effects. Therefore, careful evaluation of the soil parameters surrounding the pile foundation warrants further investigation when developing a more general three-dimensional numerical model.

This method combines a three-dimensional numerical simulation of the pile cap–pile group–soil system and machine learning techniques.

## Figures and Tables

**Table 1:** Soil parameters around pile foundation [40].

Name of Soil Layer	Thickness/m	Density/(kg·m^−3^)	Force of Cohesion/(kPa)	Angle of Internal Friction	Poisson Ratio	Dynamic Elastic Modulus/MPa
Plain fill	3	1800	28.8	24.2	0.32	121
Mild clay	4.1	2050	83	18.4	0.41	215
Silt	1.9	1930	9	31.1	0.31	180
Mild clay	16	2050	83	18.4	0.41	215
Pebble bed	3	2000	32.5	31.3	0.26	230
Clay	∞	2200	50.2	20.5	0.40	449

**Table 2:** Accuracy comparison of various machine learning techniques for predicting vertical static stiffness.

Algorithm Type	Optimal Function	RMSE	R^2^	MSE	MAE	Frequency of Training
Linear regression	Robust regression	0.036	0.93	0.001	0.025	2959
Decision tree	Subdivision tree	0.121	0.34	0.014	0.088	2456
SVM	Secondary SVM	0.066	0.91	0.004	0.05	6323
Gaussian process regression	Matern 3/2 GPR	0.032	0.95	0.001	0.020	3856
Hybrid tree	Boosted tree	0.157	0.11	0.024	0.133	4400

**Table 3:** Accuracy comparison of several machine learning models for predicting vertical dynamic stiffness.

Algorithm Type	Optimal Function	RMSE	R^2^	MSE	MAE	Frequency of Training
Linear regression	Robust regression	0.106	0.77	0.0111	0.089	1621
Decision tree	Subdivision tree	0.153	0.51	0.0233	0.130	2163
SVM	Secondary SVM	0.066	0.91	0.004	0.05	6323
Gaussian process regression	Matern 3/2 GPR	0.053	0.94	0.003	0.035	3268
Hybrid tree	Boosted tree	0.214	0.03	0.045	0.17	2891

**Table 4:** Comparison of the algorithm accuracy for predicting vertical static stiffness.

Algorithm Type	Optimal Function	RMSE	R^2^	MSE	MAE	Frequency of Training
Linear regression	Linear regression	0.097	0.78	0.009	0.074	1660
Decision tree	Subdivision tree	0.164	0.38	0.027	0.131	1890
SVM	Secondary SVMR	0.062	0.92	0.004	0.048	1855
Gaussian process regression	Exponential Gaussian	0.084	0.84	0.007	0.061	5568
Hybrid tree	Boosted tree	0.200	0.07	0.04	0.16	1485

## Data Availability

The data presented in this study are available on request from the corresponding author. The data are not publicly available due to restrictions on the subjects’ agreement.
